# Verletzte Geburtsintegrität während der COVID-19-Pandemie in Deutschland: Erfahrungen von Gebärenden mit der geburtshilflichen Versorgung

**DOI:** 10.1007/s00103-023-03667-7

**Published:** 2023-02-08

**Authors:** Stephanie Batram-Zantvoort, Anita Alaze, Marzia Lazzerini, Emanuelle Pessa Valente, Ilaria Mariani, Benedetta Covi, Céline Miani

**Affiliations:** 1 AG Epidemiologie und International Public Health, Fakultät für Gesundheitswissenschaften, Universität Bielefeld, Universität Str. 25, 33615 Bielefeld, Deutschland; 2 Institute for Maternal and Child Health IRCCS Burlo Garofolo, WHO Collaborating Centre for Maternal and Child Health, Trieste, Italien; 3 Sexual and Reproductive Health and Rights Research Unit, Institut National d’Études Démographiques (INED), Aubervilliers, Frankreich

**Keywords:** Geburtsintegrität, Geburtshilfe, Geburtserfahrung, Geburtserleben, COVID-19-Pandemie, Birth integrity, Maternity care, Experiences of care, Birth experience, COVID-19 pandemic

## Abstract

**Einleitung:**

Die COVID-19-Pandemie könnte die Vulnerabilität von Frauen gegenüber einer Verletzung ihrer Integrität während der Geburt verstärken. In einer Querschnittsstudie (März 2020 bis März 2022) wurde untersucht, wie Gebärende die Geburtshilfe während der Pandemie in Deutschland erlebten und welche Faktoren mit Geburtsintegrität assoziiert sind.

**Methodik:**

In der Befragung (validierter Fragebogen und zwei offene Fragen) beschrieben Frauen ≥ 18 Jahre ihre Erfahrungen mit geburtshilflicher Versorgung. Die quantitative Auswertung erfolgte durch deskriptive Statistik und logistische Regressionsanalysen zum Zusammenhang von Erfahrungen mit geburtshilflicher Versorgung und dem Gefühl, würdevoll behandelt und emotional unterstützt zu sein, gedeutet als gewahrte Geburtsintegrität. Die offenen Fragen wurden mittels qualitativer induktiver Inhaltsanalyse analysiert.

**Ergebnisse:**

Daten von 1271 Gebärenden und 214 Kommentare wurden ausgewertet. Die Mehrheit fühlte sich emotional unterstützt (71 %) und mit Würde behandelt (76 %). Ein Drittel gab an, manchmal oder nie in die Entscheidungsfindung involviert worden zu sein, 14 % sahen sich körperlichen, verbalen oder emotionalen Übergriffen ausgesetzt. Für 57 % der Frauen war die Begleitperson nur begrenzt oder nicht anwesend. Diese Faktoren sind alle mit der Chance assoziiert, sich würdevoll behandelt und emotional unterstützt zu fühlen. Die qualitativen Kommentare geben Aufschluss darüber, was Frauen als integritätsverletzend wahrnehmen.

**Diskussion:**

Während der COVID-19-Pandemie zeigt sich die Vulnerabilität von Gebärenden gegenüber Integritätsverletzungen. Maßnahmen zur Förderung einer respektvollen Geburtshilfe umfassen strukturelle und politische Lösungen sowie Forschung zu weiteren Determinanten von Geburtsintegrität.

## Abstract (en)

**Introduction:**

The COVID-19 pandemic may increase women’s vulnerability through violations of their integrity during birth. In a cross-sectional study (March 2020 to March 2022), we investigated how women giving birth experienced maternity care during the pandemic in Germany and which factors were associated with their birth integrity.

**Methods:**

In a survey (validated questionnaire and two open-ended questions), women ≥ 18 years described their experiences of maternity care. We conducted quantitative analyses using descriptive statistics and logistic regressions to investigate factors associated with dignified care and emotional support, which are understood as proxies of birth integrity. We analysed the open-ended questions through inductive content analysis.

**Results:**

We included data from 1271 participants and 214 comments. The majority of respondents felt emotionally supported (71%) and treated with dignity (76%). One third reported not always being involved in decision-making, while 14% felt they were subjected to physical, verbal or emotional abuse. For 57% of women, their companion of choice was absent or their presence limited. Those factors were all associated with the chances of feeling treated with dignity and emotionally supported. The qualitative comments provided an insight into what specifically women perceive as violating their integrity.

**Discussion:**

During the COVID-19 pandemic, the vulnerability of parturients lies in the violation of their birth integrity. Measures to promote respectful maternity care of women who give birth include comprehensive structural and political solutions as well as further research on the determinants of birth integrity.

## Einleitung

Schwangerschaft, Geburt und die Zeit im Wochenbett sind Phasen erhöhter körperlicher und emotionaler Vulnerabilität (Verletzbarkeit) für die Gebärende [1, 2]. „Geburtsintegrität“ bezeichnet in diesem Kontext die Unversehrtheit der Gebärenden. Sie betrifft das subjektive Erleben der Geburt (Gefühle, Emotionen, körperliche Reaktionen, Deutungen) und wird von verschiedenen Determinanten beeinflusst [3, 4]. Die Wahrung von Geburtsintegrität verstehen wir als Grundrecht einer jeden gebärenden Frau^1^ in Anlehnung an die „Respectful Maternity Care Charter“ der internationalen White Ribbon Alliance [5], die Resolution des Council of Europe zu geburtshilflicher und gynäkologischer Gewalt [6] sowie die Empfehlungen der Weltgesundheitsorganisation (WHO) zur geburtshilflichen Versorgung [7] und zur Eliminierung von Respektlosigkeit und Gewalt in der Geburtshilfe [8]. Dabei können verschiedene Faktoren zum Schutz oder zur Verletzung der Geburtsintegrität beitragen.

Integritätsschützend wirken sich vor allem die positive Beziehungsqualität und das Vertrauen zur Hebamme und den Ärzt*innen aus [9, 10]. Dies können die Informations- und Wissensasymmetrie zwischen der Frau und den geburtshilflichen Fachkräften abmildern, was zum Beispiel im Konzept „Women-centered Care“ berücksichtigt wird [11]. Das Erleben, respektvoll behandelt [9, 10] und kontinuierlich versorgt zu werden [12], in Entscheidungsprozesse eingebunden zu sein [13], die Erfahrung von Achtung vor der eigenen Autonomie und den Wünschen [14], Vertrauen in die Gesundheitskräfte zu haben und sich sicher und aufgehoben zu fühlen [15] – all diese Faktoren tragen ebenfalls zur Wahrung der Geburtsintegrität bei. Eine Begleitperson der Wahl stärkt die Gebärende emotional und hat einen positiven Einfluss auf den Geburtsverlauf (verringerter Bedarf an Schmerzmitteln, Stärkung des Selbstvertrauens und Förderung der Kommunikation von Wünschen; [16]).

Eine Verletzung der Geburtsintegrität liegt vor, wenn die Frau sich nicht ernst genommen und nicht würdevoll behandelt fühlt, geburtshilfliche Entscheidungen ohne ihre ausdrückliche Zustimmung erfolgen [17] oder sie emotionale, verbale oder körperliche Grenzüberschreitungen bis hin zu Gewalt erfährt [18, 19]. Mögliche Folgen von verletzter Integrität reichen von „Geburtstrauma“ mit posttraumatischen Stresssymptomen [20, 21] über postpartale Depressionen [22] bis hin zu einer verringerten Bindung zwischen Mutter und Kind [23] und einer verminderten Wahrscheinlichkeit des Stillens [24].

Die COVID-19-Pandemie hat die ohnehin hohe Vulnerabilität von Gebärenden gegenüber einer Verletzung ihrer Geburtsintegrität verstärkt [25] und stellt einen Risikofaktor für die Missachtung von Rechten und für geburtshilfliche Gewalt dar [26]. Mit der Einschränkung von Begleitpersonen zur Geburt betraf eine der ersten Maßnahmen zur Eindämmung der Pandemie Gebärende und ihre Partner*innen unmittelbar: Obwohl die Umsetzung dieser Maßnahme von Klinik zu Klinik unterschiedlich gehandhabt wurde, erlebten viele Schwangere Zeiten, in denen sie ohne die Begleitperson ihrer Wahl in den Wehen lagen oder sogar gebaren [27, 28]. Personalausfälle, etwa aufgrund eigener Infektionen oder zusätzlicher Familienaufgaben, verstärkten die schon präpandemisch defizitären personellen Ressourcen in Geburtskliniken. Dadurch wurden die Qualität und Dauer der Kommunikation zwischen den Gebärenden und dem geburtshilflichen Personal weiter eingeschränkt [29].

Vor diesem Hintergrund erscheint es umso relevanter, die geburtshilfliche Versorgungssituation aus der subjektiven Perspektive von Frauen mit Gebärerfahrung während der COVID-19-Pandemie wissenschaftlich zu evaluieren. Im Rahmen des Mehrländerprojekts „Improving Maternal and Newborn Care in Europe“ (IMAgiNE EURO) wurde zu Beginn der COVID-19-Pandemie in 18 Ländern eine strukturierte Befragung zur Wahrnehmung der Geburtshilfe aus Sicht der Gebärenden und des Gesundheitspersonals durchgeführt [30]. Der vorliegende Artikel zielt darauf ab, die IMAgiNE-EURO-Daten für Frauen, die seit März 2020 in Deutschland geboren haben, mit Blick auf die Wahrung oder Verletzung ihrer Geburtsintegrität hin zu untersuchen. Konkret möchten wir verstehen, welche Faktoren und/oder Situationen mit einem als negativ empfundenen Geburtserleben assoziiert sind.

## Methoden

Für den vorliegenden Artikel wurde eine Mixed-Methods-Analyse der auf Deutschland bezogenen Daten der IMAgiNE-EURO-Querschnittsstudie durchgeführt.

Der Fragebogen wurde von einem internationalen Expert*innenteam entwickelt und validiert [30]. Anschließend wurde die englischsprachige Grundbefragung ins Deutsche übersetzt und ins Englische rückübersetzt [31]. Die europaweite Rekrutierung von Teilnehmenden erfolgte durch die internationalen Partner*innen aus dem IMAgiNE-EURO-Netzwerk unter Verwendung von einheitlich gestalteten Rekrutierungsmaterialien.

Die Teilnehmenden wählten selbst aus, in welcher der 24 zur Verfügung stehenden Sprachen sie an der Studie teilnehmen wollten. Sie wurden über die Studienzwecke, Datenschutzbestimmungen, Ziel und Zweck der Studie aufgeklärt und gaben ihr Einverständnis zur anonymen Teilnahme. Es wurde über das Recht aufgeklärt, eine Teilnahme an der Studie zu verweigern, ohne dass hierdurch Nachteile entstehen. Eine Incentivierung fand nicht statt.

Teilnahmekriterien waren eine Geburt in einer geburtshilflichen Einrichtung (Krankenhaus, Klinik, Geburtshaus) ab dem 01.03.2020 sowie ein Mindestalter von 18 Jahren. In der aktiven Disseminationsphase (Oktober 2020 bis März 2021) wurde der Link zur Online-Befragung über die Befragungssoftware Redcap hauptsächlich über Social-Media-Plattformen (Instagram, Twitter, Facebook) und die Netzwerke relevanter Interessengruppen (Hebammenverband, Elterninitiative, bekannte Elternblogger*innen) verbreitet.

Das Erhebungsinstrument umfasst 40 Variablen zu den Themen Bereitstellung von und Erfahrungen mit der geburtshilflichen Versorgung, Verfügbarkeit von personellen und materiellen Ressourcen und organisatorische Veränderungen der geburtshilflichen Versorgung, die als Folgen der COVID-19-Pandemie auftreten können [30]. Darüber hinaus wurden neben geburtsbezogenen und sozioökonomischen Variablen auch zwei offene Fragen mit Kommentarfeldern gestellt. Die erste Frage richtete sich an jene Frauen, die zuvor angegeben hatten, während ihres Aufenthalts in der Geburtsklinik einem körperlichen, verbalen oder emotionalen Übergriff ausgesetzt gewesen zu sein:

„Bitte spezifizieren Sie, welcher Art von Übergriff Sie ausgesetzt waren.“

Die Befragung endete mit der zweiten offenen Frage:

„Haben Sie Vorschläge zur Verbesserung der Qualität der Betreuung in der Einrichtung, in der Sie entbunden haben, oder zur Verbesserung dieses Fragebogens?“

Für die Datenanalyse haben wir eine Auswahl an Variablen verwendet, die die Geburtsintegrität und ihre Determinanten abbilden. Zudem wurden geburtsbezogene und sozioökonomische Daten sowie die Antworten auf die offenen Fragen ausgewertet.

### Quantitative Datenanalyse

Die quantitativen Daten wurden nach standardisierten Auswertungsprozessen bereinigt und auf interne Konsistenz überprüft. Fälle, bei denen 20 % oder mehr der Antworten fehlten, wurden ausgeschlossen. Zusammenfassende Statistiken und wichtige Aspekte der Geburtshilfe wurden als absolute Häufigkeiten und Prozente dargestellt. Zusätzlich wurden zwei logistische Regressionsanalysen durchgeführt, um die Assoziationen zwischen möglichen Determinanten von erhaltender vs. verletzter Geburtsintegrität zu analysieren.

Als Indikatoren für erhaltende vs. verletzte Geburtsintegrität wurden als abhängige Variablen die subjektive Wahrnehmung, würdevoll behandelt worden zu sein, und das Erleben, emotionale Unterstützung erhalten zu haben, verwendet. Die Antwortkategorien beider Variablen wurden dichotomisiert („ja“ vs. „manchmal/nein“). Als Kovariaten wurden der Geburtsmodus und eine Kombination aus Variablen ausgewählt, die respektvolle geburtshilfliche Versorgung messen und für alle Geburtsmodi verfügbar sind: Erhalt von unmittelbarer Aufmerksamkeit bei Bedarf, Erhalt einer klaren, verständlichen und persönlichen Kommunikation, Einbezug in Behandlungsoptionen und -entscheidungen und Begleitperson der Wahl anwesend wie gewünscht. Als weiterer potenzieller Einflussfaktor für verletzte Geburtsintegrität wurde eine Variable gewählt, die geburtshilfliche Gewalt widerspiegelt: Erleben eines körperlichen, verbalen oder emotionalen Übergriffs. Zwei soziodemografische Variablen (Bildung, Teilnehmerin in Deutschland geboren) und eine geburtsbezogene Variable (Parität (1 vs. > 1)) wurden darüber hinaus als Kontrollen einbezogen.

Die statistischen Analysen wurden mit Stata/SE Version 14.0 und der R‑Software Version 3.6.1 durchgeführt.

### Qualitative Datenanalyse

Die Antworten auf die offenen Fragen wurden für eine qualitative, induktive Inhaltsanalyse herangezogen [32]. Nach der Analyse wurden die Zitate auf Grundlage ihrer Aussagekraft mit Blick auf die Gesamtergebnisse ausgewählt. Wenn Rechtschreib‑, Grammatik- oder Interpunktionsfehler die Lesbarkeit des Zitats erschwerten, wurden diese korrigiert, ohne den Aussagegehalt der Kommentare zu verändern.

## Ergebnisse

### Stichprobe

An der Befragung nahmen 1271 Frauen aus der gesamten Bundesrepublik teil, wobei Nordrhein-Westfalen (29,6 %), Bayern (16,1 %) und Niedersachsen (10,0 %) den höchsten Anteil aufweisen. Knapp 15 % der Teilnehmerinnen gaben an, außerhalb Deutschlands geboren zu sein (Tab. 1). Die am häufigsten vertretene Altersgruppe ist mit 44,2 % 31–35 Jahre alt. Das Sample weist mit 38,5 % Masterabsolventinnen (oder ein vergleichbarer bzw. höherer Abschluss) ein hohes Bildungsniveau auf. Mit Blick auf die geburtsbezogenen Angaben berichteten 46,5 %, Erstgebärende zu sein. Hinsichtlich des Geburtsmodus waren spontan-vaginale („natürliche“) Geburten am häufigsten vertreten (64,4 %), gefolgt von ungeplanten Kaiserschnitten (17,5 %), geplanten Kaiserschnitten (9,7 %) und vaginal-operativen Geburten (8,4 %; Geburt unter Verwendung einer Geburtszange oder Saugglocke).

**Table 1:** 

Variablen	Ausprägungen	*n* (%)
Jahr der Geburt	2020	1103 (86,8)
	2021	128 (10,1)
	2022	5 (0,4)
	Fehlende Angabe	35 (2,7)
Bundesland	Baden-Württemberg	110 (8,7)
	Bayern	205 (16,1)
	Berlin	101 (7,9)
	Brandenburg	25 (2,0)
	Bremen	11 (0,9)
	Hamburg	31 (2,4)
	Hessen	93 (7,3)
	Mecklenburg-Vorpommern	9 (0,7)
	Niedersachsen	127 (10,0)
	Nordrhein-Westfalen	376 (29,6)
	Rheinland-Pfalz	49 (3,9)
	Saarland	14 (1,1)
	Sachsen	41 (3,2)
	Sachsen-Anhalt	19 (1,5)
	Schleswig-Holstein	32 (2,5)
	Thüringen	28 (2,2)
Teilnehmerin in Deutschland geboren	Ja	1055 (83,0)
	Nein	188 (14,8)
	Fehlende Angabe	28 (2,2)
Alter (in Jahren)	18–24	47 (3,7)
	25–30	324 (25,5)
	31–35	562 (44,2)
	36–39	240 (18,9)
	≥ 40	70 (5,5)
	Fehlende Angabe	28 (2,2)
Höchster Bildungsabschluss^a^	Kein/Grundschule/Mittlere Reife	207 (16,3)
	Abitur, Fachabitur	289 (22,7)
	Bachelorabschluss, Vordiplom, o. Ä.	258 (20,3)
	Masterabschluss, Diplom, Promotion, PhD, o. Ä.	489 (38,5)
	Fehlende Angabe	28 (2,2)
Parität	1	591 (46,5)
	> 1	652 (51,3)
	Fehlende Angabe	28 (2,2)
Geburtsmodus	Spontane vaginale Geburt	818 (64,4)
	Vaginal-operative Geburt	107 (8,4)
	Ungeplanter Kaiserschnitt	223 (17,5)
	Geplanter Kaiserschnitt	123 (9,7)

^a^ Formulierung des Bildungsniveaus, auf die sich die Partner*innen während des Delphi-Tests geeinigt haben; Übersetzung und Rückübersetzung des Fragebogens gemäß den ISPOR Task Force for Translation and Cultural Adaptation Principles of Good Practice [31]

### Quantitative Ergebnisse

Mit Blick auf die Geburtsprozesse und das Geburtsmanagement zeigt sich in Tab. 2, dass 70,7 % aller Gebärenden angaben, bei vaginalen Untersuchungen immer vorab um Einwilligung gebeten worden zu sein. Bei Frauen mit vaginal-operativer Geburt wurde bei 62,6 % vor Durchführung des Eingriffs nicht um Einwilligung gebeten und zwei Drittel dieser Frauen (67,3 %) gaben an, dass bei ihnen zusätzlich „kristellert“ wurde (starker Druck auf den Oberbauch zur Beschleunigung der Geburt).

**Table 2:** 

Geburtsprozess und Geburtsmanagement	*n* (%)
*Schmerzlinderung erbeten und/oder erhalten?*^a^	
Ja, ich habe darum gebeten und sie wurden gegeben	480/1116 (37,8)
Ja, ich habe darum gebeten, aber sie wurden mir verweigert	65/1116 (5,1)
Nein, ich habe nicht darum gebeten, aber sie wurden vom medizinischen Personal angeboten	257/1116 (20,2)
Nein, ich habe nicht darum gebeten und sie wurden mir nicht angeboten	185/1116 (14,6)
Fehlende Angabe	284/1116 (22,3)
*Nach Kaiserschnitt ausreichende Schmerzmittelgabe?*^b^	
Ja	296/346 (85,5)
Nein	50/346 (14,5)
*Uneingeschränkte Bewegung während der Wehen*^a^	
Ja, ich durfte mich bewegen	796/1116 (71,3)
Manchmal war es mir gestattet, mich frei zu bewegen	89/1116 (8,0)
Nein, ich wurde aufgefordert, die ganze Zeit im Bett zu bleiben	85/1116 (7,6)
Fehlende Angabe	146/1116 (13,1)
*Freie Wahl der Gebärposition?*^c^	
Ja	513/818 (62,7)
Manchmal	74/818 (9,0)
Nein	231/818 (28,2)
*Dammschnitt*^c^	
Ja	113/818 (13,8)
Nein	705/818 (86,2)
*Druck auf Bauch, um den Gebärvorgang zu beschleunigen?*^d^* („kristellern“)*	
Ja	72/107 (67,3)
Nein	35/107 (32,7)
*Einwilligung bei Verwendung von Geburtszange/Saugglocke*^d^	
Ja	40/107 (37,4)
Nein	67/107 (62,6)
*Einwilligung vor vaginalen Untersuchungen*	
Ja, immer	898 (70,6)
Manchmal	193 (15,2)
Nein, niemals	110 (8,6)
Ich hatte nie vaginale Untersuchungen	70 (5,6)
*Haut-zu-Haut-Kontakt mit Kind*	
Ja	1152 (90,6)
Nein	36 (2,8)
Diese Möglichkeit hatte ich aufgrund meiner Gesundheit oder der Gesundheit meines Babys nicht	83 (6,5)
*Stillversuch nach Geburt*	
Ja	1085 (85,4)
Nein	86 (6,8)
Diese Möglichkeit hatte ich aufgrund meiner Gesundheit oder der Gesundheit meines Babys nicht	100 (7,9)

^a^ Frauen mit Wehen und/oder Blasensprung. Die Unterscheidung zwischen den Geburtsmodi basiert auf den Leitlinien des National Institute for Health and Care Excellence (NICE; [46])

^b^ Frauen mit Kaiserschnitt

^c^ Frauen mit spontan-vaginaler Geburt

^d^ Frauen mit operativ-vaginaler Geburt

Die Erfahrungen mit und das Erleben der geburtshilflichen Versorgung sind in Tab. 3 dargestellt. Während der Geburt wurden 30,1 % aller Gebärenden lediglich manchmal oder nie in die Entscheidungsfindung involviert. Die Mehrheit aller Frauen gab an, dass das Gesundheitspersonal klar, verständlich und persönlich kommunizierte (78,5 %). Körperlichen, verbalen oder emotionalen Übergriffen durch Gesundheitskräfte sahen sich 13,9 % der Gebärenden ausgesetzt. Hiervon spezifizierten 3,0 %, dass sie körperlich gewaltsam behandelt wurden, 48,0 % wurden verbal erniedrigt, 64,4 % widerfuhren emotionaler Missbrauch und fast 17 % gaben an, anderen Formen von Missachtung ausgesetzt gewesen zu sein. Nach der Geburt erhielten fast 30 % keine angemessene Unterstützung beim Stillen. Knapp 19 % der Gebärenden fühlten sich nur manchmal und 4,7 % nie würdevoll behandelt. Etwa ein Drittel der Befragten fühlte sich nur manchmal (18,9 %) oder nie/fast nie (9,7 %) emotional unterstützt.

**Table 3:** 

Wahrnehmung der geburtshilflichen Versorgung	*n* (%)
*Klare, verständliche und persönliche Kommunikation*	
Ja, immer/fast immer	998 (78,5)
Manchmal	241 (19,0)
Nein, nie/fast nie	32 (2,5)
*Einbezug in Behandlungsoptionen und -entscheidungen*	
Ja, immer/fast immer	889 (69,9)
Manchmal	300 (23,6)
Nein, nie/fast nie	82 (6,5)
*Würdevolle Behandlung*	
Ja, immer/fast immer	972 (76,5)
Manchmal	239 (18,8)
Nein, nie/fast nie	60 (4,7)
*Emotionale Unterstützung*	
Ja, immer/fast immer	908 (71,4)
Manchmal	240 (18,9)
Nein, nie/fast nie	123 (9,7)
*Unmittelbare Aufmerksamkeit*	
Ja, immer/fast immer	946 (74,4)
Manchmal	277 (21,8)
Nein, nie/fast nie	48 (3,8)
*Körperliche, verbale oder emotionale Übergriffe*	
Ja, immer/fast immer	31 (2,4)
Manchmal	146 (11,5)
Nein, nie/fast nie	1094 (86,1)
*Spezifizierung: Art des Übergriffs (Mehrfachantworten möglich)*	
Körperlicher Übergriff (wurden Sie z. B. unangemessen oder ohne vorherige Zustimmung angefasst, gestoßen, geschlagen, geohrfeigt, gekniffen, festgebunden)	38 (3,0)
Verbale Beschimpfungen (wurden Sie z. B. angeschrien, beleidigt oder wurde in unhöflichem oder abfälligem Ton mit Ihnen gesprochen)	85 (48,0)
Emotionaler Missbrauch (wurden Sie z. B. vom Gesundheitspersonal vernachlässigt, verspottet oder vergessen)	114 (64,4)
Andere	30 (16,9)
*Angemessene Unterstützung beim Stillen*	
Ja	895 (70,4)
Nein	376 (29,6)

Die pandemiespezifischen Veränderungen sind in Tab. 4 abgebildet. Für fast 37,6 % der Frauen war die gewünschte Begleitperson nie/fast nie anwesend, bei 19 % lediglich manchmal. Knapp 75 % aller Befragten empfanden die Anwesenheit des Gesundheitspersonals als ausreichend, um trotz der Pandemie angemessene Unterstützung leisten zu können.

**Table 4:** 

Geburtshilfliche Versorgung und COVID-19-Pandemie	*n* (%)
*Anwesenheit des Gesundheitspersonals ausreichend, um trotz der COVID-19-Pandemie angemessene Hilfe zu gewährleisten*	
Ja, immer/fast immer	945 (74,4)
Manchmal	261 (20,5)
Nein, nie/fast nie	65 (5,1)
*Begleitperson der Wahl anwesend wie gewünscht*	
Ja, immer/fast immer	552 (43,4)
Manchmal	241 (19,0)
Nein, nie/fast nie	478 (37,6)
Zufriedenheit mit Besuchszeiten für Partner*in und/oder Verwandte	
Ausgezeichnet/gut	339 (26,7)
Ausreichend	313 (24,6)
Unzulänglich/sehr schlecht	619 (48,7)

Die Regressionsanalysen ergaben, dass sich bei Frauen, die nicht immer/nur manchmal sofortige Aufmerksamkeit erhielten, mit denen nicht immer/nur manchmal klar, verständlich und persönlich kommuniziert wurde, die nicht immer/nur manchmal in Entscheidungsprozesse involviert waren, die nicht durchgängig von der Person ihrer Wahl begleitet wurden oder die immer oder manchmal körperlichen, verbalen oder emotionalen Übergriffen ausgesetzt waren, die Chancen signifikant verringern, sich sowohl würdevoll behandelt als auch emotional unterstützt zu fühlen, im Vergleich zu jenen Frauen, die dies immer bzw. nie erlebten (Tab. 5). Dabei zeigt das Erleben von körperlichen, verbalen oder emotionalen Übergriffen eine starke Assoziation mit verletzter Geburtsintegrität (würdevolle Behandlung: OR 0,11; 95 %-KI 0,07–0,18; emotionale Unterstützung: OR 0,21; 95 %-KI 0,14–0,33) im Vergleich zu Frauen, die sich nicht übergriffig behandelt fühlten.

**Table 5:** 

Variablen	Würdevolle Behandlung			Emotionale Unterstützung		
	OR	95 %-KI	*p*-Wert	OR	95 %-KI	*p*-Wert
*Unmittelbare Aufmerksamkeit*						
Ja, immer/fast immer	1	–	–	1	–	–
Nie/fast nie/manchmal	0,36	0,23–0,56	< 0,001	0,61	0,41–0,89	0,011
*Klare, verständliche und persönliche Kommunikation*						
Ja, immer/fast immer	1	–	–	1	–	–
Nie/fast nie/manchmal	0,18	0,11–0,28	< 0,001	0,51	0,34–0,78	0,002
*Einbezug in Behandlungsoptionen und -entscheidungen*						
Ja, immer/fast immer	1	–	–	1	–	–
Nie/fast nie/manchmal	0,26	0,17–0,39	< 0,001	0,41	0,29–0,57	< 0,001
*Körperliche, verbale oder emotionale Übergriffe*						
Nein	1	–	–	1	–	–
Ja	0,11	0,07–0,18	< 0,001	0,21	0,14–0,33	< 0,001
*Begleitperson der Wahl anwesend wie gewünscht*						
Ja, immer/fast immer	1	–	–	1	–	–
Nie/fast nie/manchmal	0,26	0,16–0,4	< 0,001	0,53	0,38–0,72	< 0,001
*Geburtsmodus*						
Spontane vaginale Geburt	1	–	–	1	–	–
Vaginal-operative Geburt	0,75	0,37–1,52	0,425	0,59	0,35–0,99	0,047
Ungeplanter Kaiserschnitt	0,56	0,34–0,92	0,023	0,39	0,27–0,57	< 0,001
Geplanter Kaiserschnitt	1,57	0,77–3,17	0,214	1,05	0,62–1,8	0,848
*Alter (in Jahren)*						
≤ 30	1	–	–	1	–	–
31–35	1,54	0,97–2,44	0,07	1,27	0,88–1,82	0,196
≥ 40	1,4	0,78–2,5	0,261	1,3	0,83–2,03	0,244
*Höchster Bildungsabschluss*						
Kein/Grundschule/Mittlere Reife	1	–	–	1	–	–
Abitur, Fachabitur	2,43	1,34–4,41	0,003	1,36	0,83–2,21	0,224
Bachelorabschluss, Vordiplom, o. Ä.	2,17	1,17–4,05	0,015	0,74	0,45–1,21	0,226
Masterabschluss, Diplom, Promotion, PhD. o. Ä.	2,82	1,6–4,97	< 0,001	0,83	0,53–1,31	0,43
*Parität*						
1	1	–	–	1	–	–
> 1	0,82	0,53–1,27	0,367	0,94	0,67–1,31	0,719
*Teilnehmerin in Deutschland geboren*						
Ja	1	–	–	1	–	–
Nein	1,15	0,65–2,06	0,631	1,29	0,83–2,00	0,257

*OR* Odds Ratio, *KI* Konfidenzintervall

Frauen mit mindestens (Fach‑)Abitur haben eine 2‑mal so hohe Chance, sich würdevoll behandelt zu fühlen im Vergleich zu Frauen mit niedrigeren Bildungsabschlüssen. Mit Blick auf die Geburtsmodi zeigt sich, dass Frauen mit einem ungeplanten Kaiserschnitt im Vergleich zu jenen mit spontan-vaginalen Geburten eine verringerte Chance haben, sich sowohl würdevoll behandelt als auch emotional unterstützt zu fühlen (würdevolle Behandlung: OR 0,56; 95 %-KI 0,34–0,92; emotionale Unterstützung: OR 0,39; 95 %-KI 0,27–0,57). Frauen mit einer vaginal-operativen Geburt haben im Vergleich zu jenen mit spontan-vaginalen Geburten eine verringerte Chance, sich emotional unterstützt zu fühlen (OR: 0,59; 95 %-KI 0,35–0,99).

### Qualitative Ergebnisse

Rund ein Viertel aller Teilnehmerinnen teilte ihre Gedanken im offenen Kommentarfeld mit (*n* = 279). Davon sind die Kommentare ausgeschlossen, die nicht auf Deutsch sind (*n* = 20) und weitere 45 Kommentare, die sich auf die Umfrage selbst bezogen. Die übrigen 214 Kommentare beschrieben das Geburtserleben bzw. die Erfahrungen mit der geburtshilflichen Versorgung und wurden in die Analyse einbezogen.

Das Gefühl verletzter Geburtsintegrität wurde explizit oder implizit in vielen Kommentaren zum Ausdruck gebracht. Dabei schilderten die Frauen ihr Erleben unterschiedlicher Situationen, die sich auf die Maßnahmen zur Eindämmung der COVID-19-Pandemie zurückführen lassen oder sich allgemein auf die geburtshilfliche Versorgungsqualität beziehen.

Die durch die COVID-19-Maßnahmen induzierte Abwesenheit oder eingeschränkte Anwesenheit der Begleitperson (meist Partner*in) wurde als ungerechtfertigt und herausfordernd wahrgenommen, belastete das Wohlbefinden und wirkte sich negativ auf das Geburtserleben der Gebärenden aus (*n* = 41). Die restriktive Begleitregelung beschrieben Frauen als emotional verletzend bis hin zu traumatisierend:

„Ist nicht schön allein in einem kalten Raum zu sein, gerade wenn es zu Komplikationen kommt. … Ich muss echt sagen, die Geburt in dieser Form hat mich echt traumatisiert …“ (TN1341)

„Mein Partner hat mir sehr gefehlt und er wurde vom Personal sehr schlecht behandelt. Ich wollte ihn dabei haben und dieser Wunsch wurde nicht respektiert. … Mein Mann und ich leiden noch immer, …, so menschenunwürdig.“ (TN77)

Das Gefühl des Alleinseins verstärkte sich durch die mangelnde Unterstützung oder fehlende Anwesenheit der geburtshilflichen Fachkräfte und des Pflegepersonals auf der Geburts- und Wochenbettstation (*n* = 41):

„Die psychische Belastung durch das Alleinsein … in den frühen Wehenphasen und in den ersten Tagen nach der Geburt war … stark belastend und teilweise traumatisierend.“ (TN1256)

„Es ist eine sehr schwierige Situation, wenn man alleine in den Kreißsaal muss ohne Partner, unter starken Wehen mit schwerer Tasche in den 4. Stock muss ohne Hilfe. Auf der Station liegen, ohne zusätzliches Personal und ohne Unterstützung vom Partner … Situation war sehr belastend …“ (TN192)

„Lag von oben bis unten vollgekotzt im Kreißsaal, da ich keine Hilfe holen konnte. Ich kam nämlich nicht an die Klingel um Hilfe zu holen, als es mir schlecht ging.“ (TN1733)

Darüber hinaus zeugen die Kommentare von Personalmangel und daraus entstehenden Arbeitsbelastungen, die sich in Stress und Überforderung der Fachkräfte oder unzureichender Unterstützung, vor allem beim Stillen (*n* = 15), äußerten:

„Ich habe mich während den Wehen nicht durch das Personal unterstützt gefühlt. Frauen sollten während der Geburt durch die Hebammen begleitet werden und nicht nur alle Stunde die Werte gecheckt bekommen und medizinische Eingriffe vorgeschlagen bekommen.“ (TN73)

„Auf der Wöchnerinnenstation hat eindeutig Personal gefehlt. Das Personal war bemüht, stand aber offensichtlich unter Stress und … nicht in der Lage, den Bedürfnissen der Patientinnen gerecht zu werden.“ (TN465)

„Ich hätte mir mehr Hilfestellung beim Stillen gewünscht. Auf Fragen zum Stillen wurde teilweise mit wenig Verständnis/Empathie reagiert …“ (TN1461)

In einigen Kommentaren konstatierten Frauen, dass sie auf der Geburtsstation übergriffig und respektlos behandelt, (verbal) gedemütigt oder ihrer Autonomie beraubt wurden.

„Ein Arzt hatte mir gesagt ich soll nicht mehr schwanger sein …, so unfreundlich … diese Ärztin hat mich so runtergemacht, so demütigend, so habe ich mich gefühlt.“ (TN159)

„Autonomie, RESPEKT, ich habe nichts dergleichen erfahren. Hätte ich nicht mein Baby bekommen … würde ich mir das Leben nehmen. Es war der schlimmste Tag meines Lebens als mein größtes Wunder zu mir kam.“ (TN237)

Andere beschrieben konkret, wie ihnen körperliche Gewalt angetan wurde. Einige Teilnehmerinnen nannten den Kristeller-Handgriff (TN35, TN329) und schmerzhafte vaginale Untersuchungen (TN412), bei anderen wurden Geburtsverletzungen ohne (ausreichende) Betäubung genäht oder der Kaiserschnitt ohne wirksame Narkose durchgeführt.

„Durch einen Dammschnitt musste ich genäht werden. Die Ärztin hat dafür 1,5 Stunden gebraucht. Mir sind die Beine eingeschlafen und ich habe keine Betäubung erhalten!“ (TN621)

„Keine ausreichende Analgesie während Kaiserschnitt. Keiner hat reagiert, bis ich ohnmächtig wurde.“ (TN2541)

„OP ohne Narkose, obwohl ich mitteilte, dass ich alles spüre. ‚Wir müssen jetzt anfangen‘, so die Ärztin.“ (TN2478)

Diese Gebärenden erlebten nicht nur körperlich unnötige Untersuchungen, sondern auch Demütigung:

„Mehrfache vaginale Untersuchungen durch mindestens fünf Hebammen, weil sie ‚sowas noch nie getastet haben‘ …, auch gegen starke Wehen. Aufforderung, auf den Boden zu urinieren, statt zur Toilette zu gehen (wäre noch möglich gewesen).“ (TN2512)

„Untersuchungen waren gewaltvoll, ich wurde gedemütigt, ich wurde ausgelacht.“ (TN1373)

Respektlosigkeit zeigte sich auch in Form von Ignoranz, Drängen zu Interventionen (TN1542), verspotten, nicht ernst nehmen („Schmerzen wurden heruntergespielt“ (TN2540)) oder der Aussage, dass man sich nicht so „anstellen soll“ (TN600):

„… als ich klar äußerte, was ich will, wurde mir gesagt, ich habe mich zu fügen und alles hinzunehmen.“ (TN307)

„Nötigung zu unnötigen Interventionen durch ständigen Druck der Hebammen. Keine Erklärung.“ (TN602)

„Nach der Geburt wurde mir vorgeworfen, nicht kooperiert zu haben und deswegen Schuld an den Verletzungen zu sein, ich musste betteln, dass diese nochmal untersucht wurden.“ (TN919)

Wiederum andere berichteten, dass ohne vorherigen Konsens Medikamente verabreicht wurden:

„Es wurde mir gegen meinen Willen … eine wehenfördernde Tablette vaginal gegeben.“ (TN1048)

„… heimlich Medikamente ohne Zustimmung in Infusion gespritzt.“ (TN35)

## Diskussion

Diese Mixed-Methods-Analyse der Daten unserer Querschnittsstudie berichtet über die Geburtserfahrungen und das -erleben von 1271 Frauen, die während der COVID-19-Pandemie in geburtshilflichen Einrichtungen in ganz Deutschland geboren haben. Unsere Auswertungen zeigen, welche Einflussfaktoren die Vulnerabilität von Gebärenden gegenüber einer verletzten Geburtsintegrität erhöhen.

Als ein direkt auf die Pandemie zurückzuführender Einflussfaktor auf Geburtsintegrität zeigt die Einschränkung von Begleitpersonen eine verringerte Chance, sich würdevoll behandelt und emotional unterstützt zu fühlen. Die Auswertung der Kommentare unterstreicht, wie belastend die Gebärenden die Zeit ohne ihre Begleitperson erlebten und Gefühle von Hilflosigkeit und Alleinsein durchlebten. Beschreibt eine Frau ihre Geburt rückblickend als traumatisch, so kann dies als Indikator für verletzte Geburtsintegrität gedeutet werden. Mit diesem Ergebnis reiht unsere Studie sich in Erkenntnisse aus europäischen Nachbarländern [33–35] und den Vereinigten Staaten [36] ein. Die Limitierung der Begleitpersonen war von Beginn der Pandemie an eine der umstrittensten, aber auch am konsequentesten umgesetzten Maßnahmen in geburtshilflichen Einrichtungen. Dabei hat die Forschung schon lange vor der Pandemie gezeigt, wie positiv sich die Anwesenheit einer Begleitperson auf den Geburtsvorgang, geburtsspezifische Outcomes (z. B. Geburtsdauer und -modus) und eine positive Geburtserfahrung auswirkt [12, 16, 37]. Viele Verbände sowie die WHO haben flexiblere Begleit‑, Zugangs- und Besuchsregeln gefordert [38]. Dennoch wurden Begleitpersonen (zumindest teilweise) von den Gebärstationen ausgeschlossen, was den jahrelangen Maßnahmen des öffentlichen Gesundheitswesens zur Förderung der Rolle von Begleitpersonen als wesentliche Akteure bei der Geburt entgegensteht [39].

Besonders belastend empfanden Teilnehmerinnen unserer Studie die Situation, weder eine Begleitperson zu haben noch ausreichend Unterstützung durch Hebammen oder das Wochenbettpersonal erfahren zu haben, wie in den Zitaten zum Ausdruck gebracht wird. Dass die Erfahrung, keine unmittelbare Aufmerksamkeit durch Hebammen und Ärzt*innen zu erhalten, die Chancen erhöht, sich nicht würdevoll behandelt und emotional unterstützt zu fühlen, konnte ebenfalls aufgezeigt werden. Obwohl unsere quantitativen Daten keinen Vergleich zwischen den wahrgenommenen Geburtserfahrungen und dem Geburtserleben vor und während der Pandemie zulassen, geben die qualitativen Kommentare dahingehend Einblicke und zeugen davon, dass die der geburtshilflichen Versorgungssituation inhärenten Probleme auch in der Pandemie präsent sind und vermutlich verstärkt wurden.

Insbesondere die bereits vor der Pandemie regelmäßig medial und politisch diskutierte sowie wissenschaftlich fundierte Personalknappheit [40] scheint sich zum Nachteil aller Beteiligten intensiviert zu haben. In Übereinstimmung mit Studien [41] und Berichten von Zeitungen und Berufsverbänden bemerkten die Teilnehmerinnen dieser Studie, dass (insbesondere) die Hebammen aufgrund voller Stationen bei zu wenig Personal unter enormem Stress und Zeitdruck standen. Diese personelle Unterbesetzung zeigte sich gleichermaßen auf den Wochenbettstationen (defizitäre Unterstützung beim Stillen und in der Versorgung des Neugeborenen), wobei die Stillberatung bereits vor der Pandemie als verbesserungsbedürftig identifiziert worden ist [42]. Die IMAgiNE-EURO-Studie hat ebenfalls eine Befragung unter geburtshilflich arbeitendem Gesundheitspersonal durchgeführt, wobei die Analyse dieser Daten (voraussichtlich 2023) ermöglichen wird, ein vollständigeres Bild der Arbeitsbedingungen des Personals in der geburtshilflichen Versorgung und auf den Wochenbettstationen zu erhalten.

Sowohl die statistischen Ergebnisse als auch die Kommentare deuten darauf hin, dass informierte Einwilligung (Konsens) und die Einbindung in Entscheidungsprozesse zu geburtshilflichen Prozeduren für Gebärende Relevanz besitzen. Die Voraussetzung hierfür liegt in einer vermittelnden, erklärenden und respektvollen Kommunikation des geburtshilflichen Personals mit der Gebärenden, besonders in Situationen, die ein zeitnahes geburtshilfliches Handeln erforderlich machen, wie sekundäre Kaiserschnitte und vaginal-operative Geburten. Frauen mit diesen Geburtsmodi zeigen in unserer Studie eine verminderte Chance, sich emotional unterstützt und würdevoll behandelt zu fühlen. Die Auswirkung von effektiver Informationsvermittlung und informierter Einwilligung auf ein positives Geburtserleben wurde unter anderem durch eine französische prospektive Kohortenstudie [43] und eine Querschnittstudie aus Polen [44] bestätigt.

In der Befragung berichteten etwa 14 % der Frauen von verbalen, körperlichen oder emotionalen Übergriffen. Die durchgeführten Regressionsanalysen zeigen, dass diese Form von Gewalt während der Geburt die Chancen, sich würdevoll behandelt und emotional unterstützt zu fühlen, deutlich verringert. Die Auswertung der Kommentare unterstreicht, wie gedemütigt und verletzt sich Frauen fühlen, die emotional, körperlich oder verbal erniedrigt wurden. In einer niederländischen Studie (*n* = 12.239) zu Missachtung und Gewalt während der Geburt auf Basis der 7 „Disrespect-and-abuse“-Kategorien nach Bohren et al. [18] gaben knapp 55 % der Teilnehmerinnen an, mindestens eine Form von körperlicher, physischer oder verbaler Missachtung erlebt zu haben (30 % physische Gewalt, 3 % emotionale Gewalt, 10 % verbale Gewalt; [45]). Während Frauen, die gar keine Gewalt erlebten, zu über 90 % eine positive Geburtserfahrung berichteten, waren es unter jenen Frauen, die eine Form von Missachtung oder Gewalt erlebten, lediglich knapp 75 %.

Die Thematik rund um Respektlosigkeit und Gewalt in der Geburtshilfe wurde inzwischen weltweit erforscht und auch in Europa und den USA belegt [19, 46, 47]. Trotzdem bleibt sie in Deutschland sowohl wissenschaftlich als auch politisch unterbelichtet. Unsere Studie trägt zur Erkenntnis bei, dass weitere Forschung unter Einbeziehung des internationalen Diskurses um respektvolle und gewaltfreie Mutterschaftsbetreuung und zum Erhalt von Geburtsintegrität dringend notwendig ist. Einerseits, um einen Überblick darüber zu erhalten, wie prävalent die Problematik in der deutschen Geburtshilfe tatsächlich ist, und andererseits, um besser zu verstehen, welche zusätzlichen Aspekte integritätserhaltend oder integritätsverletzend wirken. Weiterer Forschungsbedarf besteht auch zu den potenziellen Folgen von verletzter Geburtsintegrität [47, 48].

### Stärken und Einschränkungen

Unsere Querschnittsstudie greift auf eine relativ kleine Stichprobe von Teilnehmerinnen zurück. Wie bei dieser Art von Studien üblich, wiesen die Teilnehmerinnen einen hohen formalen Bildungsgrad auf. Dennoch entsprechen die Ost-West-Verteilung [49], der Anteil der im Ausland geborenen Frauen [49] und die Kaiserschnittrate [50] den Werten in der Allgemeinbevölkerung. Ein Selektionsfehler ist im Hinblick auf die Betreuungserfahrungen zu erwarten, wobei Frauen mit schlechteren Erfahrungen möglicherweise eher an der Studie teilgenommen haben. Dennoch überwiegt der Anteil der Frauen mit positiven Geburtserfahrungen.

### Fazit

Unsere Studie gibt Aufschluss über pandemieinduzierte bzw. verstärkte Mängel in der Geburtshilfe, die einer strukturellen und politischen Lösung bedürfen. Die interaktionalen Aspekte der Beziehung zwischen Gebärenden und den Gesundheitskräften sollten als Maßstäbe einer qualitativ hochwertigen Geburtshilfe zum Schutz der Integrität von Gebärenden als besonders vulnerable Personengruppe eingefordert und gefördert werden.

## Footnotes

1 Wir beziehen alle Personen mit eigener Gebärerfahrung ungeachtet ihrer geschlechtlichen Identität ein. Aus Gründen der einfacheren Lesbarkeit verwenden wir in diesem Artikel die Begriffe Gebärende, Frauen oder Mütter.
